# The Plight of Female Cameroonian Migrant Sex Workers in N’Djamena, Chad: A Case of Intersectionality

**DOI:** 10.1007/s10903-021-01216-5

**Published:** 2021-05-18

**Authors:** Sianga Mutola, Ngambouk Vitalis Pemunta, Ngo Valery Ngo, Ogem Irene Otang, tabi-Chama James Tabenyang

**Affiliations:** 1 School of Public Health and Community Medicine, Institute of Medicine, Sahlgrenska Academy, University of Gothenburg, Box 463, 405-30 Gothenburg, Sweden; 2 Noble Philanthropic Social Group NGO, BP 1306 Limbe, Cameroon; 3 Department of Anthropology, University of Bayreuth, Bayreuth, Germany

**Keywords:** Sex work, Intersectionality, Human rights abuse, Chad, N’Djamena

## Abstract

In most countries, sex-work is criminalized and frowned upon. This leads to human rights abuses, especially for migrant female sex workers. The burden is heavier on migrant female sex-workers whose gender and foreign citizenship intersect to produce a plethora of adverse health, social, and legal outcomes. This phenomenological study explores the intersectionality of individual factors leading to human rights abuses among migrant Cameroonian female sex workers in N’Djamena, Chad. Ten female sex workers and two key-informants were interviewed, and being a small sample, they gave detailed information about their experiences. The data was later analyzed using thematic analysis. Participants narrated experiences of social exclusion, exposure to diverse abuses, and health risks due to gender, immigrant status, and illegality of sex work. The experiences of female migrant sex workers, within contexts of sex work criminalization, are exacerbated by the intersectionality of these factors. Women endure several vulnerabilities in many African countries, more so when they have to survive on sex work as foreigners in a country where the act is illegal.

## Background

All over the world female migrant sex workers face the harsh reality of intersectionality daily. Their experiences are compounded by myriad factors—factors that cannot be disentangled without thinking about the interaction of gender, migration status, and the illegal status of sex work [1]. For a moment, it may appear as though their human rights are suspended and they have suddenly become less human than the rest. But then you have to ask whether this is because they are female, or because they are foreigners, or rather it is because they are involved in sex work in a country where it is illegal. The recent oil exploitation in Chad attracted job seekers not only from all over Chad but also neighboring Cameroon. Some of the job seekers were women, who turned to commercial sex work due to unemployment. This led to an upsurge in the number of sex workers on the streets of the Chadian capital, Ndjamena, to the effect that most of the estimated 1,200 sex workers [2] in Chad are not locals. They come from the north of Cameroon, a 15-minutes drive from the border town of Kousseri. Some are refugees escaping political instability and violence from either the Central African Republic since 2003 or Boko Haram violence from northern Nigeria [3].

Sex work in Chad is illegal, and according to Walker [4], migrant female sex workers face multiple vulnerabilities including sexual abuse, discrimination, criminalization, and physical violence. In most African societies, these acts against sex workers are structural and are entrenched into all systems including healthcare. Migrant sex workers face discrimination within the healthcare and education systems, challenges finding affordable and safe accommodation, and lack of access to childcare and psychological support services. Besides, they encounter widespread stigmatization and xenophobic attacks or attitudes from society, branding them as international “immoral women” and “foreigners” [1]. This highlights the intersectionality of being female, being a migrant, and selling sex where it is illegal to do so.

Like in many Sub-Saharan African countries, sex workers are an HIV high-risk group in Chad. According to the results of a survey conducted by the Chadian government, the rate of HIV infection among sex workers was 20% compared to a national prevalence rate of 3.3%. One-third of sex workers interviewed in the survey thought mosquito bites or sharing a meal could spread HIV. Although almost half the sex workers were tested, few had a clear understanding of the disease. The most misinformation was reported in the central and northern regions. The lack of understanding of the infection is compounded by low usage of condoms and poor access to healthcare due to their lack of power and control as a consequence of the intersectionality of gender, migrant status, and illegal sex work [5]. They are inordinately exposed to stressors such as HIV/AIDS, sexually transmitted infections (STIs), unwanted pregnancies, and unsafe abortions.

Barriers to healthcare, like access to testing and treatment of HIV and STIs, anti-foreigner sentiments from service providers and lack of health insurance have also been reported elsewhere [6–11]. However, most authors usually limit their investigations to only identifying the impact of sex work. This study delves into understanding how the individual factors produce societal level inequalities for female migrant sex workers. The importance of this approach is to emphasize the fact that interventions would not be successful in improving the welfare of sex workers if all factors were not considered.

## Theoretical Framework

We approach the plight of female Cameroonian migrant sex workers in N’Djamena, Chad from the point of intersectionality: which is a theoretical framework for understanding how multiple social identities; (1) gender (being a female), (2) citizenship (being a migrant), and (3) sex work (illegality) intersect at the micro-level of individual experience to reflect interlocking systems of oppression (sexual abuse, social exclusion, criminalization, denied access to health care) at the macro social-structural level [1]. We elicited female migrant sex workers’ daily lived experiences in their sex work, interacted with the society and the health care system to understand how the three individual factors acted together to compound their experiences. Unlike most studies on sex work whose focus is usually on a single dimension such as gender, migration, or illegal sex work, this study brings all the three dimensions together and casts a spotlight on how they affect migrant female sex workers. This approach generates an understanding that the challenges faced by migrant female sex workers, as a minority group, do not only emanate from any one of these dimensions but a result of their intersection. This, therefore, entails that for any interventions to be successful, they must address all the dimensions.

## Methods

### Participants and Data collection

A phenomenological approach was used to analyze interviews collected among women from Cameroon in N’Djamena the capital city of Chad from February 2013 to December 2013. A phenomenological approach is a type of qualitative research theory and practice [12]. In-depth interviews were used to collect data from participants by giving accounts of their experiences because this method is suitable to collect rich, personal experiences [12]. The use of open-ended questions gave the participants latitude to freely provide as much information about their experiences in sex work as possible. Through a casual, circumstantial selection process and snowball sampling system, ten (10) sex workers (aged 21 to 30) from Cameroon volunteered to participate in the study. Initial contacts were made through two (2) bartenders and a pimp (a gentleman who linked sex workers to customers and vice versa, at a fee) whom we had befriended during our regular weekend visits to the bars. The bartenders, one of whom claimed to be a “supplier” of women from Cameroon, were subsequently interviewed as key informants because they are daily witnesses to the sex work and sometimes, also served as pimps to the women and their clients. Given the hidden nature of selling sex in a criminalized and highly stigmatized context, directly locating participants who were willing to participate was not easy and required offering incentives such as drinks to sex workers to compensate for the time lost. Most of them preferred to receive money equivalent to the cost of a drink (USD 2) for one interview session. These once-off individual-in-depth interviews that lasted for between 20 and 45 minutes each were conducted at the residences of the sex workers by two male members of the research team with training in social anthropology and sociology and with extensive research experience on sensitive topics, also competent in both the French and English languages. The interviews and other research conversations were conducted in French which is the *lingua franca* and widely spoken language in the study site. Using an interview guide, we explored their demographics, how and why they came to Chad, their experiences with their clients (remuneration and working conditions), as well as their experiences with the police and the healthcare providers. Participants had the latitude to deviate from the interview or to answer the questions in any other way appropriate, as well as to disengage from the interviews with no repercussions. Additionally, interviewees were free to respond to questions or to clarify unclear phenomena weeks after their interviews. The data was recorded by the interviewers by writing down responses in notebooks as well as by audio recordings after each participant gave consent to voice recording. The interviewers exercised high levels of professionalism, patience, and caution in handling each of the participants with respect and dignity to avoid jeopardizing their safety and security considering the sensitivity of the topic. This helped the two male interviewers to build trust with the participants who were oftentimes insecure and expressed doubt to avoid being abused by men.

A major methodological challenge in studying this vulnerable minority group was their relative inaccessibility. This explains why we limited our snow-ball sampling to 10 sex workers. This also obliged us to recruit the sex workers through a pimp and the two bartenders who were willing to participate in the study. Most of the sex workers were hesitant to participate due to the illegal nature of sex work and the desire to protect their identities. However, the data abstracted from the 10 participants was adequate to answer the research questions as saturation was reached; no new information was emerging by the tenth interviewee.

### Measurements and Data Analysis

The data were anonymized and analyzed according to thematic analysis [13]. Two of the research team members were competent in both English and French languages and this facilitated for easy translation of the interview notes from French to English. The interview scripts were read several times by each of the research team members, including the interviewers, within 24 hours of the interview to search for statements or phrases with meanings regarding the participants’ experiences in respect to their female gender, foreign status, and the illegal sex work they were involved in. The voice records were also transcribed into verbatim on paper. The combination of written notes and voice recording allowed us to capture all of the participants’ spoken responses. We moved back and forth between the written transcripts and the voice notes to make sure that no detail was left out. The responses by the participants were sorted and grouped into meaning units which were answering research questions regarding the impact of gender, migrant status, and being involved in illegal sex work and how these intersected to produce different human rights abuses perpetrated by men, the police, and healthcare workers. We coded the condensed meanings into codes representing similar ideas mainly answering why the participants started sex work; what they were experiencing due to the illegal status of sex work in Chad; and how being female and migrants affected their experiences in sex work as well as in their interactions within the community. The final themes were: (1) the commodification of the female body; (2) abused for being migrants; (3) and enduring crimes for committing “crime”. We repeated the above process by exchanging the interview transcripts among us to make sure that we had a consensus over the themes. These constituted the main findings of this study.

## Results

This study aimed at exploring the intersectionality of individual factors: female gender; migrant status; and illegal sex work, to document how they produce human rights abuses at the macro-level drawing from the experiences of migrant Cameroonian female sex workers in N'Djamena, Chad. After the data was carefully analyzed, three main themes emerged, namely: the commodification of the female body; abused for being migrants; and enduring crimes for committing “crime”. Below we outline the study findings according to the themes.

## The Commodification of the Female Body

All the interviewed sex workers acknowledged that sex work helped them to escape severe poverty and that it had given them financial independence. This was in response to the question of how and why they joined sex work. The majority of the respondents expressed displeasure in having to engage in sex work under circumstances where they could not negotiate for safer sex because of the unequal power dynamics that favored the men and subjugated the women. They complained about the difficulties they faced because they bore the female gender. Recounting how she ended up joining the sex work, one participant stated that:

*“I came to the road because my parents left me when I was still very young at the age of 16 years. Because I had no real education, I had no one to take care of me so I resorted to using my body somewhere very few people knew me. But I feel exploited being a woman and most of my clients abuse me by raping, beating, or not paying me after sex because I am a woman.”* (Case No. 1)

The lack of alternative sources of income caused most sex workers to engage in sex work. Furthermore, the respondent added that being female placed her in a vulnerable position of exploitation and abuse. This is a very important finding of the study in which the participant explains that one of the reasons why men abused the sex workers was because they (men) felt more powerful than women. Men sometimes raped, beat, and refused to pay them mainly because the sex workers had no matching physical masculinity to fight back or defend themselves because of being female. The above finding also brings into perspective the Fundamental Cause Theory by Bruce Link and Jo Phelan which claims that socioeconomic status places people at “risk of risks” [14]. The lack of employment led many of the women into the sex work but ended up being abused as one college graduate narrated:

*“I graduated with a degree in Marketing from Soa, University of Yaoundé II but couldn’t find a job. So, I decided to come here to use my body but I am usually taken advantage of, being female, I can’t fight back when being abused.”* (Case No. 6)

Indeed, there are several reasons why women resort to sex work but clearly, poverty resulting from gender inequalities was the main reason why most of the respondents engaged in sex work, while the desire to generate income to supply personal and family needs kept them resilient in doing what they were doing despite the bad experiences.

## Abused for Being Migrants

The participants narrated about the bad experiences they faced due to their immigrant status. This theme emerged quite strongly in the participants’ narratives. They complained about being abused purely because of being immigrants and that they felt powerless to set the terms for their service to the clients because they were living in a foreign country. This was in addition to their female gender that placed them under the men’s dominion. Here we found that the female gender intersected with the immigrant status of the sex workers to exacerbate their vulnerability to all forms of human rights abuses. They described developing survival strategies, like using drugs to cope with the stress. One participant complained that:

*“…standing by the road for men in a foreign country is very dangerous. I usually have to act under the influence of hard drugs like exol, tramadol, diazepam… to evade police officers and also cope with being called an international prostitute.”* (Case No. 1)

The use of drugs to make themselves brave, shameless, and resilient was a phenomenon re-echoed by almost all the participants. They took these drugs to brave the cold and scares of the nights as they faced strange men every night as a coping mechanism other than finding comfort and solace in each other. They stated that the drugs had bad withdrawal effects such as tremors and lethargy and as a result, most of the sex workers had become addicted to the drugs.

Another participant stated that:

*“As a result of being denied condoms at the clinic for being a foreigner, I have to keep aborting. I sometimes have vaginal itches but the nurses are very mean so I take herbs. Maybe if I was a man, my voice would be heard when I request for any services”* (Case No. 2)

The statement was common, expressing the inequalities faced by the sex workers primarily because of being migrants. Sadly, according to the respondent above, sex workers were denied access to health services because of being immigrants and females. This finding is striking because it reveals how gender and minority status amplify the inequalities and abuses perpetrated against this group even at the public health facilities in Chad. The respondent adds that she would use herbs to treat vaginal infections, a method that may not be safe and/or efficacious. The foregoing may entail that due to lack of access to safe and effective health services, sex workers may be a reservoir for STIs and HIV and the main transmitters of these infections to the general public. This is because the sex workers, as a minority group in Chad, have been neglected. Furthermore, the theme of being exploited by their clients because of being female and holding less societal power to negotiate for safer sex was very common among the responses. They complained about how they were treated by some of their clients. Some men treated them literally like a purchased commodity after paying for sex. One participant narrated her experience as follows:

*“My body once on the street is treated like a utility which men use as they wish. Sometimes if I try to negotiate for safer sex, they beat me leaving my face swollen and bleeding. I have to cope with all these men because I am a foreign woman and I cannot report them anywhere.”* (Case No. 7)

The above sentiments about sex workers being beaten up by men and abused because they were migrant female sex workers were confirmed by one of the key informants who stated that the sex workers were always pursued by the police and that they were always at risk of being arrested, denied treatment at the health facilities, and being beaten up by their clients. The barman said:

*“I mediate between clients and the girls. Some men are aggressive and beat the girls after using them because they know that they are foreigners. Some girls who are HIV positive tell me and I help them to access anti-retro-viral drugs (ARVs)”* (Barman)

The above information confirms that men perpetrate violence against female sex workers by taking advantage of their gender, immigrant status, and the illegality of sex work to the extent that barmen would mediate and help resolve some of the conflicts.

## Enduring Crimes for Committing “Crime”

All of the respondents stated that they sometimes got raped by their clients, some policemen, or were not paid for sexual encounters because there were no laws that protected them from the standpoint of the female gender, being migrants, and practicing sex work which is illegal in Chad. Most of the participants lamented that in addition to their female gender and immigrant status, the criminalization of sex work in Chad made them more vulnerable to human rights abuses, including but not limited to, physical assault, illegal arrests, gang-raping, non-payment for sex, forced oral and/or anal sex leaving them physically bruised and emotionally traumatized. The above experiences were compounded by denied health services such contraception, condoms and STIs treatment at health facilities, and stigma and discrimination in the community. One respondent stated to that effect as follows:

*“We depend on ourselves, even when we are violated, we are the ones to be arrested. There isn’t even a need to report the police because that means turning oneself in.”* (Case No. 10)

Another participant confirmed the occurrence of physical violence in the course of sex work occasioned by the police.

*“I was once picked up by two police officers who took turns in raping me in their car, stating that I could not report anywhere because I was just a prostitute.”* (Case No. 7)

Another one recounted as followed:

*“The law is our biggest enemy; we are not protected. We end up being abused by any man and if we refuse, they threaten to take us to the police. One day I was arrested and had to use all the money I had to bribe the police officers for my release.”* (Case No. 10)

The findings outlined above indicate that migrant sex workers in Chad were a neglected minority group whose plight does not seem to concern the government because of the lack of legal frameworks and policy to protect them. The participants in this study recounted how they experienced different types of abuse due to their female gender, their foreign citizenship, and the criminalization of sex work in Chad; dimensions that leave the sex workers vulnerable to the human rights violation. The human rights violations included lack of access to healthcare services, physical violence, rape, extortion, illegal arrests, and psychological torment.

## Discussion

Sex workers in most African countries face diverse forms of abuse from all sections of society. The experiences of sex workers chronicled in this paper demonstrate an intersection of gender, foreign citizenship, and the illegal status of sex work in Chad. Unlike Scorgie et al. [15] who argue that bad attitudes about sex work are fuelled by the rise in conservative religious fundamentalism, we posit that; female sex workers are more likely to face sexual abuse than their male counterparts, migrant female sex workers will face worse human rights abuses than their local counterparts, and female migrant sex workers operating in a country where sex work is a crime will face the worst human rights abuses. In this sense, the findings of this study have shown that the combination of the three identities increases the gravity of human rights abuses inflicted on sex workers and magnifies their vulnerability to inequalities in accessing healthcare services and/or receiving legal protection.

The International Organization of Migration (IOM) acknowledges that the migration of people and the resultant health implications are gendered phenomena. This means that women, men, children, and sexual minorities face different socioeconomic and health risks before, during, and after migration [16]. It is also true that despite the wide recognition of migration-related risks by governments, like the government of Chad, migrants often face punitive policies, arbitrary detentions, sex and physical abuses, extortion, and denied access to health services in the pretext of “national security concerns” [17]. The foregoing is in tandem with the findings of this study which show that the female gender, the immigrant status, and the illegal status of sex work intersect and are taken advantage of to perpetrate human rights violations against sex workers. This is against the tenets promulgated by the United Nations with regards to the right to health which entitles “the enjoyment of the highest attainable standard of health conducive to living a life in dignity regardless of one’s race, color, creed, nationality, gender, or sexual orientation” [18]. In line with Campbell’s [19] argument concerning the importance of holistic approaches in understanding the intersection of several factors operating together against the welfare of sex workers elsewhere, we also strongly believe that the three factors highlighted in this paper operate in unison to produce several human rights cases of abuse against sex workers in Chad. Of particular interest is the fact that sex workers in Chad are discriminated against from having access to either basic or tailored healthcare services. Therefore, the government or indeed any other change agency may not be successful in ameliorating the plight of sex workers without paying attention to the impact of gender and power imbalances between men and women, actively protecting the rights of migrants, and decriminalizing sex work. This is based on the findings of this study showing that the three factors intersect and should not be dealt with in isolation.

Additionally, McBride et al. [20]**,** convincingly posit that immigrant sex workers face disproportional human rights abuses compared to their local counterparts since being a foreigner imparts a social status less than that of the citizens of that particular country, putting the foreign sex workers at increased vulnerability to human rights abuse. Furthermore, the criminalization of sex work impedes efforts to reach sex workers with social services such as tailored health services, and legal protection against abuses and violence. The illegal nature of sex work is taken advantage of by clients, the police, and the community, weaponizing it for non-payment after sex, rape, verbal and physical violence, and arrests [15]. As reported in this study, there is systemically entrench stigma and discrimination against sex work in Chad, such that even professionally trained health workers verbally abuse sex workers (compounded by foreign citizenship) and alienate them from accessing psychosocial counseling, treatment for STIs, and/or access.

Based on the above arguments, we recommend policy reforms in Chad that will ensure a paradigm shift in terms of how migrant female sex workers, as a minority group, are treated. Firstly, since women’s sexual rights are largely ignored and abused by men making them vulnerable to sexual abuses [15] including in Chad, the government must recognize women and gender rights and enforce laws that protect women in general and sex workers in particular. Secondly, the government, through the Ministry of Justice and the Ministry of Public Security and Immigration, should ensure a seamless process for migrant documentation and set regulations that protect them from discrimination based on country of origin. This is because immigrant sex workers face disproportional human rights abuses compared to their local counterparts [15]. Additionally, the government through the Ministry of Public Health and National Solidarity should commit to the provision of health services to all according to the principles of the Right to Health [18] and engage all other stakeholders, such as Non-governmental organizations and Civil Society Organizations, in bringing health services to migrant female sex workers. Thirdly, the criminalization of sex work impedes efforts to reach sex workers with social services such as tailored health services, and legal protection against abuses and violence. The criminalization of sex work is taken advantage of by clients, the police, and the community for non-payment after sex, rape, verbal and physical violence against the sex workers [20]. Therefore, the government of Chad should embark on a process to decriminalize sex work. We acknowledge that the process of actuating the above recommendations may be lengthy and controversial to some stakeholders, but we suggest that it may be helpful for all stakeholders to engage in honesty reflections and conversations about the legal, social, and health implications of continued neglect of this minority group.

## New Contribution to the Literature

The approach of the intersectionality of the micro-level individual factors and how they compound the abuses experienced by migrant female sex workers in a criminalized context that this study adopted brings in novel information. Additionally, utilizing phenomenology ensured abstraction of detailed information with regards to lived experiences which forms a solid foundation for further research, policy development, and/or design of public health interventions. From a public health standpoint, the three highlighted individual-level factors must be holistically addressed, failure to which human rights violations against migrant female sex workers will perpetuate. Furthermore, the fight against STIs and HIV is incomplete in the absence of programs targeted at sex workers, who are disproportionally more affected by these diseases than the general public.

## Conclusion

This study demonstrates the intersectionality of gender, migrant citizenship, and illegal status of sex work, and how they work in unison to produce human rights abuses at the macro-level against Cameroonian migrant female sex workers in Chad. Sex workers are raped, beaten, and abused by men, arrested and detained by the police, as well as denied healthcare services because of gender imbalance and power dynamics around sex, being an immigrant, and lack of legal protection. The paper also suggests policies and legal reforms to ameliorate the plight of sex workers in Chad.
